# 

**Published:** 1859-01

**Authors:** 


### THE SANITARY REVIEW.

### CONTENTS of No. XVI.

Life and Labour . . . . . . . . 317

Health of England in 1856 . . . . . . . 328

**EPITOME OF SANITARY LITERATURE:**

Watson. Resources of India . . . . . 334

Armstrong. Naval Hygiene and Scurvy . . . . 33[illegible]

Dickinson. Egypt a Residence for Invalids . . . 338

Infant Hygiene . . . . . . . 339

Swimming for Women . . . . . . 340

Women's Clothing . . . . . . 341

Williams. Climate of Aspley Guise . . . . 342

**ORIGINAL COMMUNICATIONS:**

Suggestions towards a more perfect System of Self-acting Ventilation. By J. N. Radcliffe, Esq. . . . . . 343

Influence of the Seasons of the Year, Employment, etc., on the Gain or Loss of Weight by the Prisoners in the Convict Prison at Wakefield. By W. R. Milner, Esq. . . . . . 348

The Use of the Moustache and Beard in the Royal Navy. By D. J. Duigan, M.D. . . . . . . 361

Working among Rags. By J. J. Murray, Esq. . . . . 363

Fever and Diarrhœa from Foul Water. By W. I. Cox, Esq. . . 371

**SANITARY AND SOCIAL SCIENCE:**

*Reports of Cities, Towns, and Districts.*—Bombay . . . 373

Purification of the Thames . . . . . . 386

**PROGRESS OF EPIDEMICS** in **Forty-Two Towns and Districts, contributed specially, by the Staff of Local Observers** . . . 391

**SANITARY LEGISLATION:** . . . . . . 410

**TRANSACTIONS of the EPIDEMIOLOGICAL SOCIETY of LONDON:**

Introductory Address. By B. G. Babington, M.D. . . . 73

Illustrations of the Origin and Propagation of certain Epidemic Disorders. By T. H. Barker, M.D. . . . . . . 79

